# Camel milk ameliorates steatohepatitis, insulin resistance and lipid peroxidation in experimental non-alcoholic fatty liver disease

**DOI:** 10.1186/1472-6882-13-264

**Published:** 2013-10-13

**Authors:** Aida A Korish, Maha M Arafah

**Affiliations:** 1 Physiology Department (29) College of Medicine, King Saud University, PO Box 2925, Riyadh 11461, Saudi Arabia; 2 Pathology Department, College of Medicine, King Saud University, PO Box 2925, Riyadh 11461, Saudi Arabia

**Keywords:** Camel milk, Non-alcoholic fatty liver disease, Steatohepatitis, High-fat diet, Insulin resistance, Hyperlipidemia, Oxidative stress, Rats

## Abstract

**Background:**

Camel milk (CM) is gaining increasing recognition due to its beneficial effects in the control and prevention of multiple health problems. The current study aimed to investigate the effects of CM on the hepatic biochemical and cellular alterations induced by a high-fat, cholesterol-rich diet (HCD), specifically, non-alcoholic fatty liver disease (NAFLD).

**Methods:**

Seventy male Wistar rats were divided into four groups: the Control (C) Group fed a standard diet; the Control + camel milk (CCM) Group fed a standard diet and CM, the Cholesterol (Ch) Group fed a HCD with no CM, and the Cholesterol + camel milk (ChM) Group fed a HCD and CM. The following parameters were investigated in the studied groups; basal, weekly random and final fasting blood glucose levels, intraperitoneal glucose tolerance test (GTT) and insulin tolerance test (ITT), serum insulin, serum lipids, liver functions, lipid peroxidation products, the antioxidant activity of catalase (CAT) and the levels of reduced glutathione (GSH). In addition, HOMA-IR as an index of insulin resistance (IR) and the histopathology of the hepatic tissue were assessed.

**Results:**

The Ch Group developed features similar to those of non-alcoholic steatohepatitis (NASH), characterized by hepatic steatosis; inflammatory cellular infiltration in liver tissue; altered liver functions; and increased total cholesterol, triglycerides, low-density lipoprotein cholesterol, very-low-density lipoprotein cholesterol, atherogenic index (AI), blood glucose, IR, and malondialdehyde (MDA) levels. Additionally, feeding the HCD to animals in the Ch Group decreased CAT activity and the GSH and high-density lipoprotein (HDL) cholesterol levels. Camel milk intake for eight weeks decreased hepatic fat accumulation and inflammatory cellular infiltration, preserved liver function, increased the GSH levels and CAT activity, decreased the MDA levels, and ameliorated the changes in the lipid profile, AI, and IR in animals from the ChM Group.

**Conclusions:**

CM has a unique composition that is rich in minerals; vitamins, insulin and insulin-like protein, and it increased HDL-cholesterol and ameliorated the biochemical and cellular features of NAFLD in rats that received a HCD. The antioxidant effect of CM is a likely mechanism for the altered metabolism and absorption of HCD in the presence of CM. Regular consumption of CM could provide a natural way to protect against NAFLD induced by a high-fat diet.

## Background

Fatty liver refers to a large spectrum of diseases characterized by excessive fat accumulation in the liver, which could be alcoholic or non-alcoholic in origin. Non-alcoholic fatty liver disease (NAFLD) is clinically important because it affects 25% of the population, with widespread pathological changes in the liver that range from simple non-progressive steatosis to non-alcoholic steatohepatitis (NASH). This can progress to cirrhosis, hepatocellular carcinoma, and liver failure with increased hepatic-related mortality [1,2].

Clinically, NASH is linked to visceral obesity, insulin resistance (IR), dyslipidemia, and type II diabetes mellitus. The clustering of these metabolic risk factors, identified as 'insulin resistance syndrome,’ increases free fatty acid (FFA) release from adipose tissue, which results from decreased insulin-mediated repression of lipolysis, and induces NASH [3].

The relationship between IR and NASH is reciprocal, and they potentiate each other; therefore, scientists consider NASH to be the liver manifestation of metabolic syndrome [4]. Diet-induced obesity predisposes individuals to a spectrum of disorders, including metabolic syndrome, hepatic steatosis, and NASH. For that reason, a high-fat, cholesterol-rich diet (HCD) is commonly used in the experimental induction of hyperlipidemia and NAFLD [5]. Conversely, several plants, such as ginger, cinnamon, licorice, berries, plant leaves, and herb roots, in addition to animal products such as bones, hooves, skins, feathers, and milk, have long been used in traditional and alternative medicine. These substances are increasingly valued as raw materials in the preparation of modern medicine and herbal preparations [6].

According to the World Health Organization (WHO), approximately 80% of the population in some African and Asian countries and 38% in the Americas depend primarily on complementary and alternative medicine for the prevention, protection and treatment of diseases [7,8].

Camel milk (CM) is used in hot and arid regions as an essential nutritional source, and its high energy and vitamin contents are known to help immune-deficient patients as well as those recovering from diseases [9-11]. Oral CM is well tolerated by lactase-deficient children who are allergic to cow milk [12], and it shows protective effects against heavy metal toxicity [13] and viral and bacterial infections [14]. Additionally, Indians used CM for the treatment of multiple acute and chronic health problems, including asthma, anemia, jaundice, and spleen problems [15]. Interestingly, the low prevalence of diabetes in the Raica community was attributed to the regular consumption of CM [16]. This was further supported by the better glycemic control in diabetic patients and animals receiving CM [17,18].

Recent studies have shown that CM has antihypertensive, anti-cancerous, hepatoprotective, and hypocholesterolemic effects [19-22]. However, the effects of CM on HCD-induced hepatic biochemical and structural changes, oxidative-antioxidative balance and glucose homeostasis have not been investigated. The reported health benefits of CM justified the great public concern and stimulated our interest to investigate its effects on NAFLD because this conditions is one of the obesity-associated disorders that is currently widely prevalent. We hypothesized that acquiring healthy dietary habits and the regular consumption of natural products such as CM can protect against the metabolic and cellular ailments induced by a HCD, which is extensively consumed, for example, fast food meals. The present study will help the establishment of scientific background knowledge regarding CM as a natural food with potential therapeutic and protective effects against one or more currently prevalent health problems associated with dyslipidemia and IR.

## Methods

### Animals and experimental groups

Male Wistar rats, 6 to 8 wk old (weighing 270–325 g), were obtained from the Animal Care Unit of the College of Medicine, King Saud University (KSU). The Institutional Review Board (IRB) (formerly Ethical Committee) of the College of Medicine, KSU, approved the study protocol, and all of the animal handling procedures adhered to the guidelines of the College of Medicine Research Center and and the 'Guide for the Care and Use of Laboratory Animals’, as declared by the Committee on Care and Use of Laboratory Animals of the Institute of Laboratory Animal Resources, National Research Council, USA. The animals were housed five per cage in a 21°C temperature-controlled facility with a 12-h light/dark cycle and free access to rat chow and water ad libitum. After one week of acclimatization, animals were randomized into four experimental groups:

Control (C) Group (n = 10): normal rats fed standard rat chow.

Control + CM (CCM) Group (n = 20): normal rats fed standard rat chow and CM.

Cholesterol (Ch) Group (n = 20): rats fed a HCD.

Cholesterol + CM (ChM) Group (n = 20): rats fed a HCD and CM.

Body weight was recorded at the beginning and the end of the study in all the experimental groups.

### Induction of hyperlipidemia

Commercial rat chow (Grain Silos & Flour Mills Organization Riyadh Branch, Riyadh, K.S.A) that contains 20% crude protein; 4% fat; 3.5% crude fiber; 6% ash, 0.5% salt, 1% calcium, 0.6% phosphorous, 20 IU/g vitamin A, 20 IU/g vitamin D, 20 IU/kg vitamin E, and trace amounts of cobalt, copper, iodine, iron, manganese, selenium, and zinc was used in the C and CCM Groups. A high-fat, cholesterol-rich diet (HCD), in which 42% of the energy is derived from fats, was prepared by the addition of 1.5% cholesterol (Sigma Aldrich, USA) and 8% coconut oil to the basal diet [5]. The HCD was prepared every 2 days, kept at 4°C, and given to rats in the Ch and ChM Groups for 8 weeks.

### Camel milk administration

Camel milk was collected daily from a private camel farm in the middle of Riyadh, Saudi Arabia, transported to the laboratory in screw-capped bottles in cool boxes, and administered orally at a rate of 250 ml/cage/day (i.e., approximately 50 ml/rat/day) for 8 weeks to the animals in the CCM and ChM Groups.

To standardize the quality of the milk used, the milked camel and its diet were kept unchanged throughout the study. A pilot study was conducted before the start of the experimental period to estimate the average volume of CM that can be consumed per rat per day, which was found to be approximately 50 ml/24 hours.

### Blood glucose

Basal, weekly random and final fasting blood glucose levels were recorded using a drop of blood from the tail vein and an Accu-Check monitor (Roche Diagnostics, West Sussex, UK).

### Intraperitoneal glucose tolerance test (GTT) and insulin tolerance test (ITT)

To investigate the effects of CM on glucose homeostasis and insulin sensitivity in animals on normal rat chow and HCD, ten rats in each group were subjected to an i.p. GTT and an insulin tolerance test (ITT) during the last week of the study. Before the GTT, rats were fasted overnight but allowed free access to water. Baseline (t = 0) blood samples were taken to measure fasting blood glucose (FBG) before i.p. injection of D (+) - glucose (1 g/kg) dissolved in 2 ml of distilled water, and the blood glucose level was recorded after 15, 30, 60, 90, 120, and 150 minutes with an Accu–Check monitor (Roche Diagnostics, West Sussex, UK) using a drop of blood from the tail vein. The ITT was performed in overnight-fasted animals by an i.p. injection of insulin (0.75 IU/kg) [23], and the blood glucose level was checked after 0, 4, 8, 12, and 16 minutes. The values are presented as percentages of the initial blood glucose level.

### Blood and tissue sampling

After eight weeks, the animals were fasted overnight, and blood samples were collected from the retro-orbital sinus into plain test tubes under Nembutal anesthesia (50 mg/kg, i.p.) [24]. Serum was separated and stored at -80°C for biochemical analysis. Animals were sacrificed, and the livers were isolated, macroscopically examined, and weighed. A small fragment of the liver tissue was frozen in liquid nitrogen for the measurement of oxidative stress markers. The remaining liver tissue was immersed in 10% neutral-buffered formalin and reserved for histopathological examination. The frozen liver tissue was homogenized with Ultra Turax Homogenizer (Janken Kunkel IKa-Werk, Staufen, Germany) in 50 mM phosphate buffer, pH 6–7, containing 1 mM EDTA at 10,000 xg for 15 min at 4°C.

### Biochemical analysis

#### Serum insulin

Insulin was measured using the rat insulin enzyme immunoassay kit (EIA), Spi-Bio, Montigny le Bretonneux, France, according to the manufacturer’s instructions and as reported previously [25].

#### Serum lipids

Serum lipids were determined using colorimetric kits from United Diagnostics Industry, Riyadh, KSA, according to the manufacturer’s instructions [26]. The atherogenic index (AI) was calculated as total cholesterol [TC] – high-density lipoprotein cholesterol [HDL-C]/ HDL-C [27].

#### Liver function tests

The serum transaminases (AST and ALT), alkaline phosphatase (AP), gamma glutamyl transpeptidase (GAMA-GT), total protein, and albumin concentrations were measured using colorimetric kits from United Diagnostics Industry, Riyadh, KSA.

#### Lipid peroxidation products

Serum and liver tissue malondialdehyde (MDA) levels were measured as thiobarbituric acid reactive substances (TBARS) with a specific EIA kit (Cayman Chemical, Ann Arbor, MI, USA) at 530–540 nm [28].

#### Catalase (CAT) activity

A commercial kit produced by Cayman Chemical, Ann Arbor, MI, USA, was used to measure the antioxidant CAT activity in serum and liver homogenates. The method is based on the reaction of the enzyme with methanol in the presence of an optimal concentration of H_2_O_2_. The enzyme activity was defined as nanomoles of hydrogen peroxide consumed per minute [29].

#### Reduced glutathione (GSH)

Liver GSH was assayed according to Ellman’s method [30] using a glutathione assay kit (Cayman Chemical, Ann Arbor, MI, USA, Catalog No, 703002).

### Assessment of the insulin resistance

The homeostasis model assessment of insulin resistance (HOMA-IR) was calculated as [fasting blood glucose (mg/dl) x fasting serum insulin (mU/l) /405] [31].

### Histopathological examination

The fixed liver tissue samples were embedded in paraffin and cut into 3–5 μm sections and stained with hematoxylin and eosin (H&E). The histopathology of the hepatic tissue was studied using an Olympus BX51 microscope and a DP72 Camera (12 MG Pixel) to assess the presence of steatosis and inflammation. The fat accumulation pattern was classified as microvesicular, macrovesicular, or mixed, and the degree of hepatocyte involvement was graded as Grade 0: no fat in the liver, Grade 1 (mild): < 23%, Grade 2 (moderate) < 33-66%, and Grade 3 (severe): > 66% [1]. The NAFLD activity score (NAC) classification included non-steatohepatitis, borderline, zone 3 steatohepatitis, and definite steatohepatitis. The scores of each component of NAS were as follows: steatosis (0–3), lobular inflammation (0–3), ballooning (0–2) and fibrosis (0–2). A score of ≤ 4 indicates borderline steatohepatitis and > 5 is definite steatohepatitis [32].

### Statistical analysis

The results are presented as the mean ± SD and were statistically analyzed with SPSS for Windows version 18.00. Comparisons between all groups were carried out by ANOVA, and when significant, the post hoc LSD test was used to assess the different groups. The results were considered significant when p < 0.05.

## Results

### Body weight

**Figure 1:**
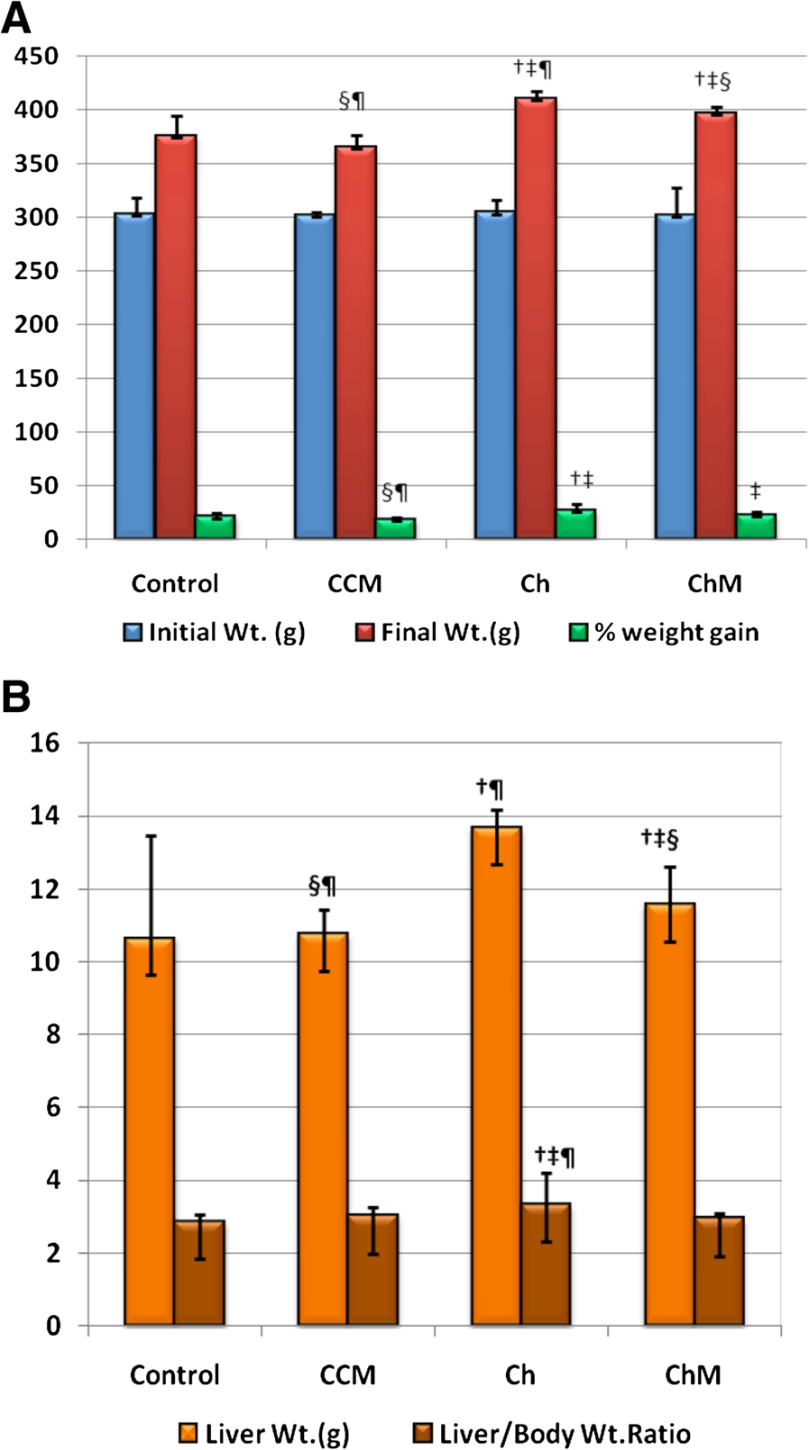
**The changes in body weight (A), liver weight and liver to body weight ratio (B) in the control and the high-fat diet groups and the effect of camel milk intake at the end of the study.** CCM; control + milk group, Ch; cholesterol group, ChM; cholesterol + milk group. Values are presented as the mean ± SD. † p < 0.05 versus control, ‡ p < 0.05 versus CCM, § p < 0.05 versus Ch, ¶ p < 0.05 versus ChM.

After 8 weeks of HCD administration, the Ch Group exhibited a significant increase in body weight compared with the C Group fed standard rat chow (p < 0.001), (Figure 1A). Camel milk significantly decreased the body weight of the ChM Group in comparison with the Ch Group (p < 0.013). Likewise, Figure 1A shows that the percentage increase in body weight at the end of the study was significantly higher in the Ch Group compared with the C Group (p < 0.01), but that the ChM Group’s weight increase was comparable with that of the C Group (p > 0.05). There was no significant difference in weight gain between the CCM and C Groups (p > 0.05).

### Liver weight and liver: body mass ratio (LBR)

Liver weight and LBR were significantly higher in the Ch Group in comparison with the C Group (p < 0.001 for both) (Figure 1B). Adding CM to the diet of the ChM Group resulted in a significant decrease in the liver weight and LBR compared with the Ch Group (p < 0.001 for each). In contrast, CM ingestion in the CCM Group did not affect their liver weight or LBR compared with the C Group (p > 0.05).

### Random blood glucose level

**Figure 2:**
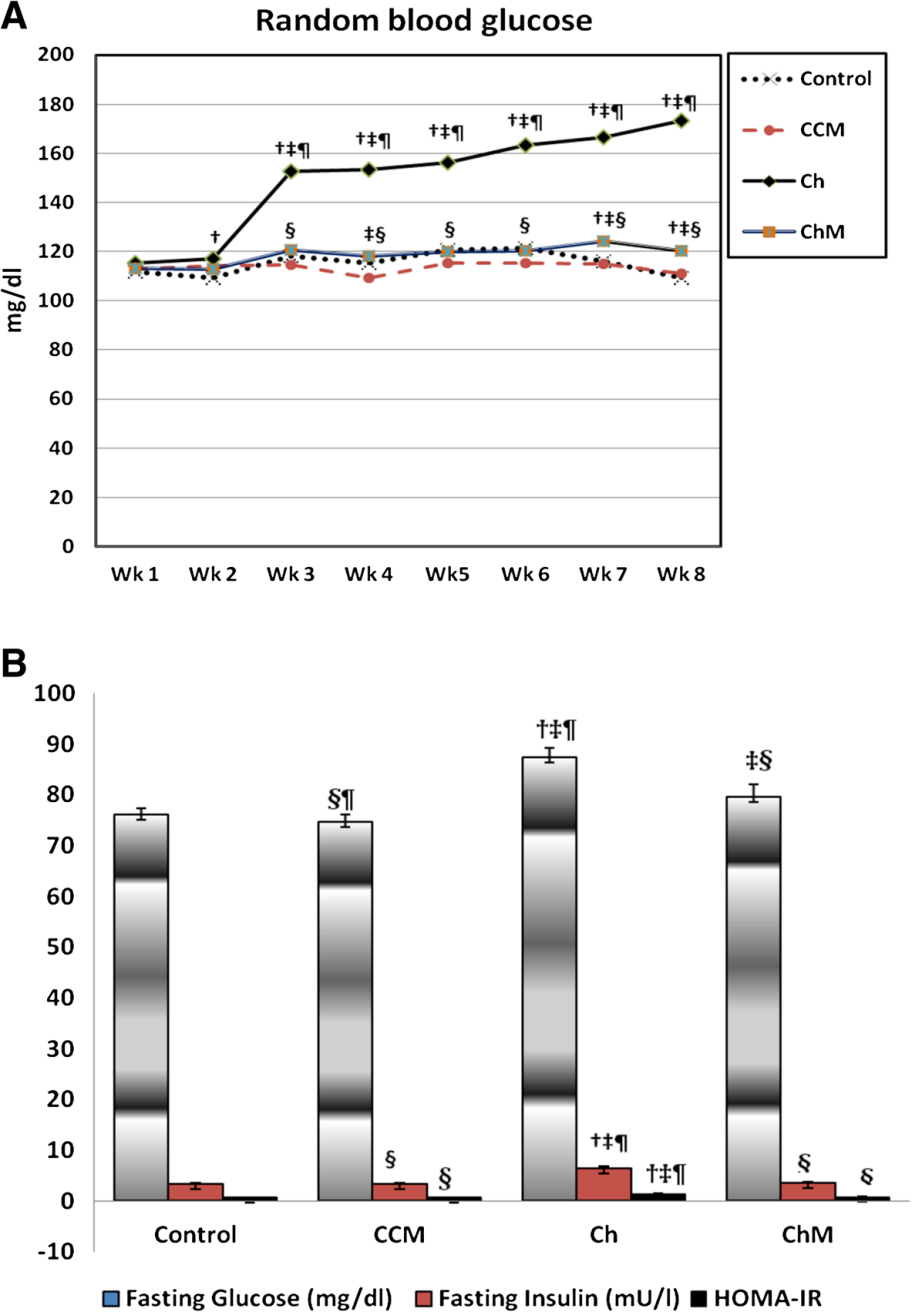
**The changes in random blood glucose level (A), fasting blood glucose, serum insulin and HOMA-IR (B) in the control and high-fat diet treated groups and the effect of camel’s milk intake.** CCM; control + milk group, Ch; cholesterol group, ChM; cholesterol + milk group. Values are presented as the mean ± SD. † p < 0.05 versus Control, ‡ p < 0.05 versus Control + camel milk, § p < 0.05 versus Cholesterol, ¶ p < 0.05 versus Cholesterol + camel milk.

After one week of receiving the HCD diet, as well as throughout the study, the Ch Group exhibited a significant increase in RBG compared with the C Group (p < 0.05) (Figure 2A). However, CM administration to the ChM Group resulted in a significant reduction in RBG in comparison to the Ch Group at each measurement (p < 0.05), with no significant difference with respect to the C Group (p > 0.05). The administration of CM to the CCM Group caused no significant change in RBG compared with the C Group (p > 0.05).

### Fasting blood glucose and serum insulin

Eight weeks of daily ingestion of HCD impaired the FBG of the Ch Group as compared with the C Group (p < 0.001) (Figure 2B). Camel milk administration inhibited HCD-induced hyperglycemia in the ChM Group in comparison with the Ch Group (p = 0.004), but there was no significant difference with respect to the C Group (p = 0.17). However, the CM-treated CCM Group rats showed no significant change in FBG compared with the C Group (p > 0.05). The fasting serum insulin level increased significantly in association with the elevated FBG in the Ch Group compared with the control Group (p < 0.001) (Figure 2B). This change was similar to that observed in patients with NASH. However, CM intake significantly decreased the fasting insulin level of the ChM Group in comparison to the Ch Group (p < 0.001), reaching the control level (p > 0.05). However, the fasting insulin level of the CCM showed no significant change in comparison with the C Group (p > 0.05) (Figure 2B).

#### HOMA-IR

A high-fat, cholesterol-rich diet induced a significant increase in the IR in the Ch Group animals compared with the control Group animals (p < 0.001), as indicated by the high HOMA-IR. Camel milk administration to the ChM Group decreased their HOMA-IR compared with the Ch Group (p < 0.001), reaching the control range (p > 0.05) (Figure 2B). Notably, the administration of CM to the CCM Group, in addition to the normal rat chow, resulted in no significant changes in the HOMA-IR compared with the C Group (p > 0.05).

#### GTT and ITT

**Figure 3:**
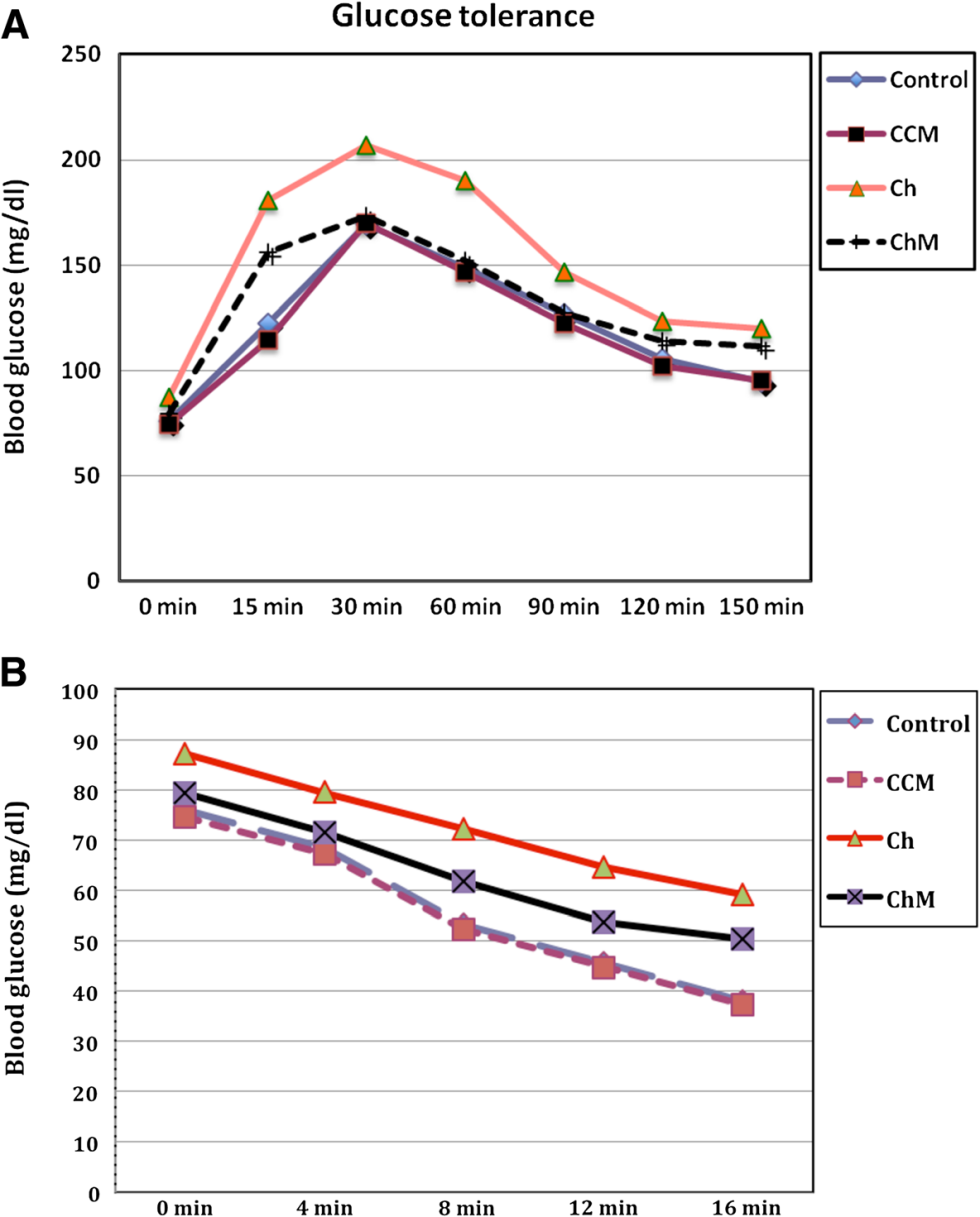
**Intraperitoneal glucose tolerance test (i.p GTT) (A), and insulin tolerance test (ITT) (B) in rats fed standard and high-fat diets with and without camel milk treatment for 8 weeks.** CCM; control + milk group, Ch; cholesterol group, ChM; cholesterol + milk group. Values are presented as the mean ± SD.

Fasting and postprandial hyperglycemia were evident in the Ch Group animals in comparison to the control Group C animals at all time points of the glucose tolerance curve (p < 0.001). The blood glucose levels in the Ch Group reached the peak at 30 min after i.p. glucose loading and then decreased slightly by 150 min but did not reach the fasting level. This confirmed the glucose intolerance (Figure 3A).

In contrast, the CM-treated ChM animals showed significant fasting hypoglycemia in comparison to Group Ch animals (p = 0.02) and the postprandial hyperglycemia was significantly inhibited, with lower levels of blood glucose in comparison to the Ch Group from 0 time until 150 min of the GTT (P < 0.05). It is worth noting that the i.p. glucose tolerance curve in Group ChM animals was comparable to that of Group C animals at 0, 30, 60 and 90 min (P < 0.05) (Figure 3A).

Intraperitoneal insulin injection caused significant comparable hypoglycemia in the C and CCM Groups during the ITT. The Ch Group showed significant inhibition in the response to insulin injection in comparison to the C Group (p < 0.001). The expected hypoglycemia after intraperitoneal injection was significantly inhibited in the CH Group compared to the C Group (the glycemic decay in response to intraperitoneal ITT in the ChM Group was significantly recovered after CM treatment as an index of the peripheral insulin sensitivity) (Figure 3B).

### Oxidative stress

The level of MDA, a marker of lipid peroxidation, increased significantly in the serum and livers of the Ch Group animals compared with the C Group (p < 0.001) (Table 1). This was associated with a significant decrease in CAT activity and GSH levels (p < 0.001). Alternatively, CM administration to the ChM Group reversed the oxidative stress effect of HCD, as evidenced by the significant reduction in the MDA level, the increase in CAT activity, and the elevation of GSH levels compared with the Ch Group (p < 0.001). The serum and liver levels of MDA, GSH, and CAT activity were comparable in both the CCM and C Groups (p > 0.05).

**Table 1:** Malondialdehyde (MDA) levels, catalase (CAT) activity and reduced glutathione (GSH) levels at the end of the study in rats fed standard and high-fat, cholesterol-rich diets with and without camel milk treatment

	**Control (n = 10)**	**CCM (n = 20)**	**Ch (n = 20)**	**ChM (n = 20)**	**F value P value**
Serum MDA μM/l	0.65 ± 0.00	0.65 ± 0.00 §	8.07 ± 1.4 †‡	0.65 ± 0.00 §	**462.02**
					**<0.001**
Liver MDA μM/g tissue	1.35 ± 0.13	1.32 ± 0.12 §	1.95 ± 0.16 †‡	1.59 ± 0.09†‡§	**47.93**
					**<0.001**
Serum CAT activity nmol H2O2/min/mg protein	5.52 ± 1.57	4.96 ± 0.61§	3.95 ± 1.04 †‡	5.02 ± 0.73 §	**17.13**
					**<0.001**
Liver CAT activity μM H2O2/min/mg protein	60.90 ± 3.72	62.51 ± 2.98 §	47.70 ± 3.37 †‡	56.30 ± 2.29 †‡§	**44.78**
					**<0.001**
Liver GSH Mg/g tissue	22.97 ± 1.13	23.11 ± 1.12 §	14.17 ± 1.38 †‡	23.11 ± 1.08 †‡§	**131.49**
					**<0.001**

CCM; control + milk group, Ch; cholesterol group, ChM; cholesterol + milk group. Values are presented as the mean ± SD. Significant differences between all groups were identified by ANOVA test presented by F value. When significant (p value < 0.05), a post hoc LSD test was performed to compare the groups. † p < 0.05 versus control, ‡ p < 0.05 versus CCM, § p < 0.05 versus Ch, p < 0.05 versus ChM.

### Lipid profile

**Figure 4:**
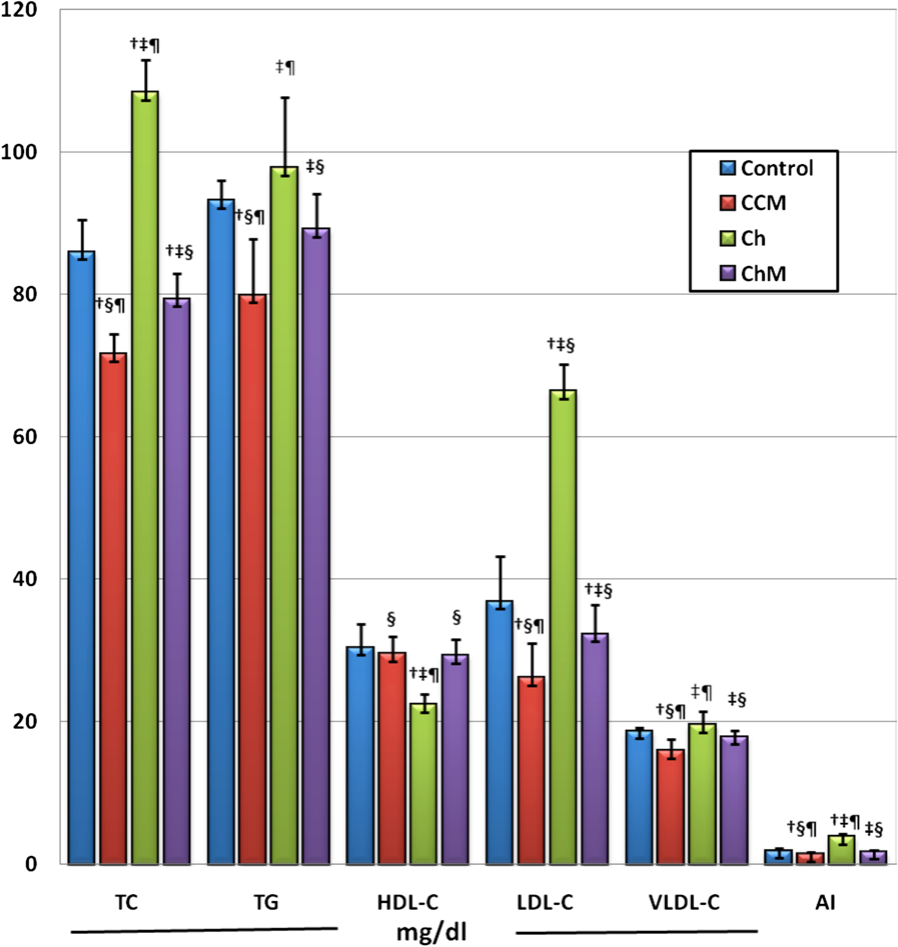
**Lipid profile and atherogenic index in rats fed standard and high-fat diets with and without camel milk treatment for 8 weeks.** CCM; control+ milk group, Ch; cholesterol group, ChM; cholesterol+ milk group. Values are presented as the mean ± SD. † p < 0.05 versus control, ‡ p < 0.05 versus CCM, § p < 0.05 versus Ch, ¶ p < 0.05 versus ChM.

The ingestion of HCD by the Ch Group animals resulted in significant increases in TC, TG, LDL-C, and VLDL-C and decreased the HDL-C levels compared with the C Group animals (p < 0.001) (Figure 4). Camel milk treatment in the ChM Group reversed the lipid profile changes induced by HCD in the Ch Group (P < 0.001), reaching the control level (p > 0.05). Interestingly, CM treatment in the CCM Group resulted in a significant reduction in the TC, TG, LDL-C and VLDL-C levels (p < 0.001) but caused no significant change in the HDL-C level compared with the C Group (p > 0.05).

### The atherogenic index (AI)

In association with the hyperlipidemia and dyslipidemia observed in the Ch Group animals, the AI was significantly higher in comparison to the C Group (p < 0.001) (Figure 4). Camel milk significantly decreased the AI in the ChM Group compared with the Ch Group (p < 0.001), reaching the control value (p > 0.05). Interestingly, CM treatment in the CCM Group induced a significant decrease in the AI in comparison with the C Group (p = 0.003). This indicates that CM had a protective effect against atherosclerosis in the control animals.

### Liver function

The administration of HCD to the Ch Group for eight weeks resulted in significant increases in the AST, ALT, AP, GAMA-GT, and bilirubin levels, but it decreased the serum protein and albumin levels compared with the C Group (p < 0.001) (Table 2). The alterations in liver functions observed in the Ch Group were abolished in the ChM Group after CM treatment (p < 0.001). Furthermore, the CCM Group showed significant elevation of the total serum protein and albumin levels compared with the C Group (p < 0.001), in the absence of any changes in the other liver function parameters (p > 0.05).

**Table 2:** Liver function tests in rats fed standard and high-fat, cholesterol-rich diets with and without camel milk treatment for 8 weeks

	**Control (n=10)**	**CCM (n=20)**	**Ch (n=20)**	**Ch M (n=20)**	**F value P value**
AST (U/L)	45.99 ± 2.47	48.16 ± 3.08 §	57.41 ± 3.18 †‡	50.91±4.18†‡§	**35.49**
					**<0.001**
ALT (U/L)	52.43 ± 2.69	54.76 ± 4.18 §	70.77 ± 4.19 †‡	56.68±3.90†‡§	**79.84**
					**<0.001**
AP (U/L)	85.39 ± 6.44	83.79 ± 6.36 §	116.33 ± 6.79 †‡	104.80 ±6.12†‡§	**105.86**
					**<0.001**
GAMA-GT (U/L)	43.47 ± 8.19	44.14 ± 6.67 §	50.12 ±5.45 †‡	45.74 ±3.95 †§	**4.46**
					**0.006**
Total bilirubin (mg/dl)	1.45 ± 0.23	1.28 ± 0.19 §	2.07 ± 0.44 †‡	1.23 ± 0.14 †§	**36.70**
					**<0.001**
Direct bilirubin (mg/dl)	0.43 ± 0.08	0.49 ± 0.11§	0.82 ± 0.10 †‡	0.46 ± 0.08 †§	**61.65**
					**<0.001**
Total proteins (mg/dl)	4.6 ± 0.19	6.25 ± 0.23 †‡	4.17 ± 0.15 †‡	5.05 ± 0.41 †‡§	**199.68**
					**<0.001**
Serum albumin (mg/dl)	3.45 ± 0.05	3.85 ± 0.40 †§	3.15 ± 0.09 †‡	3.57 ± 0.28 †‡§	**23.31** **<0.001**

CCM; control + milk group, Ch; cholesterol group, ChM; cholesterol + milk group. Values are presented as the mean ± SD. Significant differences between all groups were identified by ANOVA test presented by F value. When significant (p value < 0.05), a post hoc LSD test was performed to compare the groups. † p < 0.05 versus control, ‡ p < 0.05 versus CCM, § p < 0.05 versus Ch, ¶ p < 0.05 versus ChM.

### Histopathology

**Figure 5:**
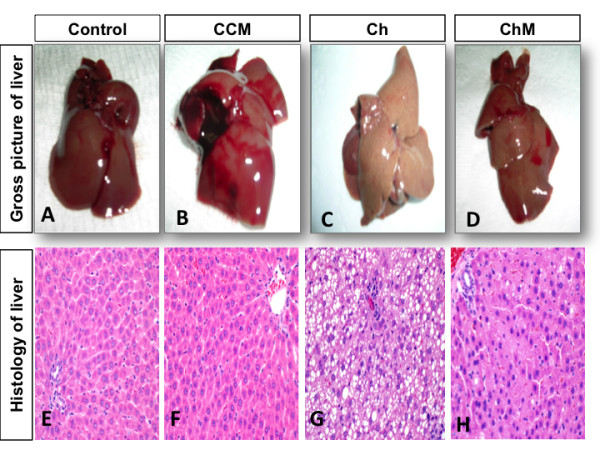
**Gross and microscopic changes in the liver of the control and high-fat diet groups with and without camel milk intake.** Part I: Gross macroscopic evaluation of the liver from the Control group **(**part **A)** and the control + camel milk Group **(**part **B)** showed red, smooth and shiny liver tissues. Livers from the Cholesterol Group **(**part **C)** that received the high-fat diet for eight weeks were extremely pale, enlarged, and extensively infiltrated with white spots that reflect fat accumulation inside the hepatic cells. The Cholesterol + milk group **(**part **D)** received camel milk for eight weeks, and the livers showed marked improvement compared to the livers of the untreated group; the livers were slightly pale with few scattered white spots compared with the Ch Group. Part II: 40X photomicrograph of the H and E stained liver tissue of the Control **(**part **E)** and the Control + milk groups **(**part **F)** showing normal polyhedral hepatocytes with a central nucleus and eosinophilic cytoplasm. In contrast, the Ch Group **(**part **G)** showed ballooning degeneration of the hepatocytes, loss of cytoplasmic eosin and eccentric nuclei, with diffuse microvesicular and macrovesicular steatosis, and foci of lobular inflammation and necrosis. Camel milk treatment in the ChM Group **(**part **H)** markedly attenuated the histopathological characteristics of NASH observed in the Ch Group. The ChM group showed only mild microvesicular steatosis, no inflammatory foci, and intact architecture.

Macroscopic examination of livers of the C and CCM Groups (Figure 5A, B) showed a normal red, smooth, and shiny appearance. In contrast, livers from the Ch Group (Figure 5C) were extremely pale, enlarged, and extensively infiltrated with white spots that reflect fat accumulation inside the hepatic cells. The livers from animals in the ChM Group receiving CM (Figure 5D) were slightly pale with few scattered white spots compared with the Ch Group. Light microscopic examination of the liver tissue in the C and CCM Groups stained by H&E showed normal polyhedral hepatocytes with central nuclei and eosinophilic cytoplasm (Figure 5E, F). In contrast, the Ch Group showed ballooning degeneration of the hepatocytes, loss of the cytoplasmic eosin, eccentric nuclei, diffuse microvesicular and macrovesicular steatosis (50% grade 1, 20% grade 2 and 30% grade 3), portal inflammation, foci of lobular inflammation, and necrosis (Figure 5G). Camel milk treatment in the ChM Group markedly attenuated the histopathological characteristics of NASH observed in the Ch Group. There was mild microvesicular steatosis in 20% of the tissues, and steatosis was nearly absent in the rest of the samples, with intact architecture and no inflammatory foci (Figure 5H).

## Discussion

The worldwide epidemic of obesity and diabetes is closely associated with the increased incidence of NAFLD [27,31]. Life style modification and the acquisition of healthy food habits are the most clinically recognized and effective methods to control the disease and minimize the progression to NASH and cirrhosis [33]. In Islamic communities, CM has gained a good reputation due to its health benefits stated by the Prophet Mohammed (PBUH) more than 1400 years ago [34] and to the folklore stories detailing its shielding effects against a wide range of diseases. Multiple recent studies have reported that CM has antiviral, antibacterial, hypoglycemic, antihypertensive, hypocholesterolemic, and anticancer activities [14,16,17,19-22].

The present study investigated the effect of CM on the metabolic alterations in the lipid profile, glucose homeostasis, IR, and oxidative-antioxidative balance and the functional and structural changes of hepatocytes in NAFLD induced by HCD. Feeding the Ch Group animals with HCD resulted in dramatic increases in the TC, TG, LDL-C, VLDL-C levels and in the AI, but it decreased the HDL-C levels. This hyperlipidemia could be related to the enhanced de-esterification of the abundant FFAs and decreased lipoproteins [35]. The biochemical picture of dyslipidemia in the Ch Group animals was associated with hepatic steatosis, focal lobular inflammation, ballooning degeneration, and necrosis of the hepatocytes, which matches the reported histopathological features of NASH [1].

In addition to hyperlipidemia, the Ch Group animals showed enhanced oxidative stress, as evidenced by increased MDA levels and decreased CAT activity and GSH levels. This could be related to the beta-oxidation of fatty acids in hepatic steatosis, which stimulates reactive oxygen species (ROS) generation, lipid peroxidation, hepatocyte necro-inflammation, and apoptosis [35]. The oxidative stress produced by excess ROS production and decreased antioxidant mechanisms plays an important role in the chronic inflammatory responses to hypercholesterolemia and is known to activate hepatic stellate cells and to trigger fibrogenesis [36,37]. Therefore, steatosis is considered the basic event in the multi-step process of NASH, which comes in association with increased total body fat and visceral obesity [38].

The adipocytes are no longer considered passive cells that store excessive triglycerides but are instead considered active cells that regulate the energy balance and secrete the pro-inflammatory cytokines Il-6, and TNF-α [39]. This leads to the suggestion that multiple immunomodulatory factors and ROS contribute to the chronic inflammatory condition and the hepatocytes injury observed in steatohepatitis [40].

The observed loss of the structural integrity of the hepatic tissue in the Ch Group animals demonstrated hepatic cell injury, which led to the release of excessive amounts of intracellular hepatic transaminases into the serum, and the high biliary pressure augmented AP, GAMA-GT, and bilirubin synthesis by the cells lining the hepatic canaliculi [41]. The structural distortion and the functional impairment of the hepatic cells by NASH in the Ch Group were associated with hyperinsulinemia, high IR, and low serum protein and albumin levels. These may be related to the excessive release of adipokines and FFAs from adipose tissue, which in turn, may induce islet cell apoptosis and liver gluconeogenesis, both of which contribute to IR, diabetes, and atherosclerotic cardiovascular complications [42].

Camel milk has a unique composition that distinguishes it from other ruminant milk. It is low in sugars, cholesterol, and proteins but is rich in minerals (sodium, potassium, iron, copper, zinc and magnesium) and vitamins (A, B2, C, and E) [43-45]. This makes it an excellent source of nutrients, vitamins, and trace elements, which have biological activities against several diseases [14,16,17,19-22].

The obvious amelioration of the hyperlipidemia and dyslipidemia in the CM-treated ChM Group is in agreement with recent reports about fresh and fermented CM containing Bifidobacteria, which lower plasma lipids in rats receiving a high-cholesterol diet [22,46]. The hypolipidemic effect of CM could be directly related to the content of high L-carnitine, which decreases cholesterol absorption [47,48]. However, two indirect mechanisms could also be proposed for the improvement of the lipid profile and the decreased body weight in the ChM Group: (i) CM may exert local effects on the stomach to inhibit gastric emptying or give a sense of satiety and decrease food intake. (ii) Camel milk may alter the PPAR alpha/SREBP1 ratio, as mentioned in recent work conducted by Ziamajidi N, et al. [49], leading to enhanced activity of the fat-metabolizing enzymes and hormones, resulting in increased caloric loss or decreased fat storage.

All of the histological features of NASH observed in the Ch Group in the present study, especially the inflammatory foci, showed marked improvement in the CM- treated ChM Group. This has a specific importance, as it was recently reported that inflammation is the only predictor of fibrosis progression and the development of cirrhosis [50]. The high magnesium and trace element contents in CM can protect against oxidative damage and help the absorption and metabolism of the antioxidant vitamins B, C, and E [45]. The antioxidant effect of CM plays a role in the reduction of hepatic fat accumulation and decreases systemic and hepatic oxidative stress [51], as evidenced by increased GSH levels and CAT activity and decreased MDA production in the ChM Group.

In addition to its specific composition, the digestion of CM in the gastrointestinal tract produces many bioactive compounds with antimicrobial, antioxidant, immunomodulatory, and hepatoprotective effects [52]. Similarly, the healing of the hepatic parenchyma observed by microscopic examination in the ChM Group was associated with the recovery of normal serum proteins and albumin levels and decreased serum transaminases, bilirubin, AP, and GAMA-GT levels. In this regard, vitamin E treatment was reported to decrease serum transaminases and hepatic steatosis [53], which supports the notion that the high vitamins and trace elements content in CM protect hepatocytes integrity and prevents the release of transaminases into the blood.

The current findings are in accordance with the similar hepatic protective effects of CM recently reported in carbon tetrachloride, paracetamol and alcohol toxicity [21,54,55]. Decreased serum insulin and RBG levels and the improved glycemic decay in response to insulin injection in the ChM Group compared to the Ch Group reflected increased peripheral response to insulin, as confirmed by the decreased HOMA-IR. The high concentration of insulin (40 units/l) and insulin-like protein and the immunoglobulin contents of CM [18,56] identified it as a natural product that not only helps glycemic control but also preserves the normal lipid profile, as evidenced in the CM-treated animals in the present study. In addition to CM, other natural products or medicinal herbs, including chicory leaves, barely grains, celery, Lyceum and Barbarum, have been reported to have both glucose-lowering and lipid-lowering capabilities [57,58].

This is the first study, to the best of our knowledge, to investigate the effects of CM on the biochemical and histological changes related to NAFLD. Camel milk exerted multi-faceted metabolic effects in HCD-induced NAFLD. These effects could be mostly attributed to the active components in CM, which may act independently or interact together in endocrine, paracrine, or autocrine modes of action, leading to the modification of the metabolic abnormalities of NAFLD [59]. However, one limitation of the current study was the difficulty in specifying the active ingredients in CM that acted either individually or through molecular interactions to produce the observed changes in NAFLD in the treated animals. The isolation and identification of such active components require a detailed bio-analytical technique that was not within the scope of the current study, but it represents an important future research area that could be investigated by our research group and other investigators.

## Conclusions

The findings of the current study led us to conclude that camel milk markedly improved the biochemical and histopathological abnormalities induced by HCD, including hyperlipidemia, steatohepatitis, impaired liver function, and insulin resistance. These findings support the reported health-promoting effects of CM and support its role in treating hyperlipidemia-associated chronic health problems resulting from unhealthy lifestyles and eating habits. However, large-scale clinical trials with large populations are still needed to confirm the results obtained from animal studies.

## Abbreviations

AChE: Acetyl cholinesterase; AP: Alkaline phosphatase; AST: Aspartate transaminase; ALT: Atherogenic index (AI), alanine transaminase; CAT: Catalase; PBUH: Peace be upon him; EIA: Enzyme immunoassay kit; FBG: Fasting blood glucose; GAMA-GT: Gamma glutamyl transpeptidase [4-glutamyl transferase; GTT: Glucose tolerance test; H&E: Hematoxylin and eosin; HDL-C: High density lipoprotein-cholesterol; HCD: High fat– cholesterol-rich diet; CM: Camel milk; HOMA-IR: Homeostasis model assessment of insulin resistance; ITT: Insulin tolerance test; LBR: Liver body mass ratio; MDA: Malondialdehyde; NAFLD: Non-alcoholic fatty liver disease; NASH: Non-alcoholic steatohepatitis; FFA: Free fatty acid; RBG: Random blood glucose; TBARS: Thiobarbituric acid reactive substances; TC: Total cholesterol; TG: Triglycerides.

## Competing interests

No competing interests are associated with study.

## Authors’ contributions

AK designed and conceived the study, collected the data, performed the statistical analysis, drafted and finalized the manuscript. MA carried out the histopathological studies and contributed to the collection of the data, interpretation of the results and editing of the paper. Both authors have approved the final version of the manuscript.

## Authors’ information

AK, Associate Professor of Physiology, Physiology Department, College of Medicine, King Saud University, Saudi Arabia and also a Professor of Physiology, Clinical Physiology Department, Faculty of Medicine, Alexandria University, Egypt. MA, Associate Professor of Pathology, Department of Pathology, College of Medicine, King Saud University, Saudi Arabia.

## Pre-publication history

The pre-publication history for this paper can be accessed here:

http://www.biomedcentral.com/1472-6882/13/264/prepub

## References

[B1] Brunt E Nonalcoholic steatohepatitis Semin Liver Dis 2004 24 3 20 15085483 10.1055/s-2004-823098

[B2] Soderberg C Stal P Askling J Glaumann H Lindberg G Marmur J Hultcrantz R Decreased survival of subjects with elevated liver function tests during a 28-year follow-up Hepatol 2010 51 595 602 10.1002/hep.23314 20014114

[B3] Cusi K Role of insulin resistance and lipotoxicity in non-alcoholic steatohepatitis Clin Liver Dis 2009 13 545 563 10.1016/j.cld.2009.07.009 19818304

[B4] Malhi H Gores GJ Molecular mechanisms of lipotoxicity in nonalcoholic fatty liver disease Semin Liver Dis 2008 28 360 369 10.1055/s-0028-1091980 18956292 PMC2908270

[B5] Mukundh NB Muralidharan P Balamurugan G Antihyperlipidemic activity of Pedalium murex (Linn) fruits on high fat diet fed rats Int J Pharmacol 2008 4 310 313 10.3923/ijp.2008.310.313

[B6] Lev E Traditional healing with animals (zootherapy): medieval to present-day Levantine practice J Ethno pharmacol 2003 86 107 118 10.1016/s0378-8741(02)00377-x 12576209

[B7] WHO, IUCN, WWF Guidelines on the Conservation of Medicinal Plants Gland 1993 Switzerland: The World Conservation Union (IUCN), in partnership with The World Health Organization (WHO) and World Wide Fund for Nature (WWF)

[B8] National Center for Complementary and Alternative Medicine (NCCAM) What Is Complementary and Alternative Medicine? National Institutes of Health 6 Feb. 2012. Web. 29 May 2012. http://nccam.nih.gov/health/whatiscam

[B9] Gorakh MD Sena DC Jain VK Sahani MS Therapeutic utility of camel milk as nutritional supplement against multiple drug resistant patients. In: Proc 2nd Intl Camelid Conf Agro economics of Camelid Farming Almaty 2000 9 99

[B10] Yateem A Balba MT Al Surrayai T Al Mutairi B Al –Daher: Isolation of lactic acid bacteria with probiotic potential from camel milk Int J Dairy Sci 2008 3 194 199 10.3923/ijds.2008.194.199

[B11] Al-Awadi FM Srikumar TS Trace elements and their distribution in protein fractions of camel milk in comparison to other commonly consumed milks J Dairy Res 2001 68 463 469 11694048 10.1017/s0022029901005003

[B12] El Agamy EI Nawar M Shamsia SM Awad S Haenlein GF Are camel milk proteins convenient to the nutrition of cow milk allergic children Small Ruminant Res 2009 82 1 6 10.1016/j.smallrumres.2008.12.016

[B13] Al-Hashem F Dallak M Bashir N Abbas M Elessa R Khalil M Al-Khateeb M Camel's milk protects against cadmium chloride induced toxicity in white albino rats Am J Pharmacol and Toxicol 2009 4 107 117

[B14] El Agamy EI Ruppanner R Ismail A Champagne CP Assaf R Antibacterial and antiviral activity of camel milk protective proteins J Dairy Res 1992 59 169 175 10.1017/S0022029900030417 1319434

[B15] Rao RB Gupta RC Dastur NN Camel’ milk and milk products Ind J Dairy Sci 1970 23 71 78

[B16] Agrawal RP Budania S Sharma P Gupta R Kochar DK Panwar RB Sahani MS Zero prevalence of diabetes in camel milk consuming Raica community of north-west Rajasthan India Diabetes Res Clin Pract 2007 76 290 296 10.1016/j.diabres.2006.09.036 17098321

[B17] Agrawal RP Swami SC Beniwal R Kochar DK Sahani MS Tuteja FC Ghouri SK Effect on camel milk on glycemic control, lipid profile and diabetes quality of life in type-1 diabetes: A randomized prospective controlled cross over study Indian J Anim Sci 2003 73 1105 1110

[B18] Al Haj OA Al Kanhal HA Compositional, technological and nutritional aspects of dromedary camel milk In Dairy J 2010 20 811 821 10.1016/j.idairyj.2010.04.003

[B19] Quan S Tsuda T Miyamoto T Angiotensin 1- converting enzyme inhibitory peptides in skim milk fermented with Lactobacillus helveticus 130B4 from camel milk in Inner Mongolia, China J Sci Food Agric 2008 88 2688 2692 10.1002/jsfa.3394

[B20] Majeed NA Corrective effect of milk of camel on some cancer biomarkers in blood of rats intoxicated with aflatoxin B J Saudi Chem Soc 2005 9 253 264

[B21] Khan AA Alzohairy MA Hepatoprotective effects of camel milk against CCl4-induced hepatotoxicity in rats in rats Asian J Biochem 2011 6 171 180

[B22] Elayan AA Sulieman AM Saleh FA The hypocholesterolemic effect of Gariss and Gariss containing bifidobacteria in rats fed on a cholesterol-enriched diet Asian J Biochem 2008 3 43 47 10.3923/ajb.2008.43.47

[B23] Ming Z Xiao-Yan L Jing L Zhi-Gang X Li C The characterization of high-fat diet and multiple low-dose streptozotocin induced type 2 diabetes rat model Exp Diabetes Res 2008 2008 2008 704 745 10.1155/2008/704045 PMC2613511 19132099

[B24] Otero P Bonet B Herrera E Robano A Development of atherosclerosis in the diabetic BALB/c mice prevention with vitamin E administration Atherosclerosis 2005 182 259 265 10.1016/j.atherosclerosis.2005.02.024 16159598

[B25] Andersen L Dinesen B Jorgensen PN Poulsen F Roder MF Enzyme immunoassay for intact human insulin in serum or plasma Clin Chem 1993 38 578 582 8472350

[B26] Friedewald WT Levy RI Fredrickson DS Estimation of the concentration of LDL cholesterol in plasma without use of the preparative ultracentrifuge Clin Chem 1972 18 499 502 4337382

[B27] Suanarunsawat T Ayutthaya WDN Songsak T Rattanamahaphoom J Anti-lipidemic actions of essential oil extracted from Ocimum sanctum L. leaves in rats fed with high cholesterol diet J Appl Biomed 2009 7 45 53

[B28] Yagi K Simple assay for the level of total lipid peroxides in serum or plasma Methods Mol Biol 1998 108 101 106 9921519 10.1385/0-89603-472-0:101

[B29] Johansson LH Borg LAH A spectrophotometric method for determination of catalase activity in small tissue samples Anal Biochem 1988 174 331 336 10.1016/0003-2697(88)90554-4 3064653

[B30] Ellman GL Tissue sulfhydryl groups Arch Biochem Biophys 1959 82 70 77 10.1016/0003-9861(59)90090-6 13650640

[B31] Matthews DR Hosker JP Rudenski AS Naylor BA Treacher DF Turner RC Homeostasis model assessment: insulin resistance and beta-cell function from fasting plasma glucose and insulin concentrations in man Diabetologia 1985 28 412 419 10.1007/BF00280883 3899825

[B32] Brunt EM Kleiner DE Wilson LA Belt P Neuschwander-Tetri BA; NASH Clinical Research Network (CRN) Nonalcoholic fatty liver disease (NAFLD) activity score and the histopathologic diagnosis in NAFLD: distinct clinicopathologic meanings Hepatology 2011 53 3 810–820 21319198 10.1002/hep.24127 PMC3079483

[B33] Xiao J Guo R Fung ML Liong EC Tipoe GL Therapeutic approaches to non-alcoholic fatty liver disease: past achievements and future challenges Hepatobiliary Pancreat Dis Int 2013 12 Suppl.2 125 135 23558065 10.1016/s1499-3872(13)60021-1

[B34] Ghiyâth Hasan al-Ahmad: Tibb al-Nabawî fî Daw' al-'Ilm al-Hadîth (2:215) and Le DROMADAIRE Un monde de soif http://www.abc.se/home/m9783/ir/f/Camel%20Milk.html (in French)

[B35] Jensen MD Role of body fat distribution and the metabolic complications of obesity J Clin Endocrinol Metab 2008 93 S57 S63 10.1210/jc.2008-1585 18987271 PMC2585758

[B36] Kojda G Harrison D Interactions between NO and reactive oxygen species: pathophysiological importance in atherosclerosis, hypertension, diabetes and heart failure Car vas Res 1999 43 562 571 10.1016/S0008-6363(99)00169-8 10690328

[B37] Feldstein AE Papouchado BG Angulo P Sanderson S Adams L Gores GJ Hepatic stellate cells and fibrosis progression in patients with nonalcoholic fatty liver disease Clin Gastroenterol Hepatol 2005 3 384 389 10.1016/S1542-3565(04)00616-0 15822044

[B38] Yamaguchi K Yang L McCall S Huang J Yu XX Pandey SK Bhanot S Monia BP Li YX Diehl AM Inhibiting triglyceride synthesis improves hepatic steatosis but exacerbates liver damage and fibrosis in obese mice with nonalcoholic steatohepatitis Hepatol 2007 45 1366 1374 10.1002/hep.21655 17476695

[B39] Schäffler A Schölmerich J Büchler C Mechanisms of disease: adipocytokines and visceral adipose tissue–emerging role in nonalcoholic fatty liver disease Nat Clin Pract Gastro- enterol Hepatol 2005 2 273 280 10.1038/ncpgasthep0186 16265231

[B40] Hui JM Hodge A Farrell GC Kench JG Kriketos A George J Beyond insulin resistance in NASH: TNF-alpha or adiponectin? Hepatol 2004 40 46 54 10.1002/hep.20280 15239085

[B41] Drotman RP Lawhorn GT Serum enzymes as indicators of chemical induced liver damage Drug Chem Toxicol 1978 1 163 171 10.3109/01480547809034433 755666

[B42] Browning JD Horton JD Molecular mediators of hepatic steatosis and liver injury J Clin Invest 2004 114 147 152 15254578 10.1172/JCI22422 PMC449757

[B43] Abu-Lehia IH Physical and chemical characteristic of camel’s milk fat and its fractions Food Chem 1989 34 261 271 10.1016/0308-8146(89)90103-9

[B44] Beg OU Bahr-Lindstrom HV Zaidi ZH Jornvall H Characterization of a camel milk protein rich in proline identifies a new beta casein fragment Regul Pept 1986 15 55 61 10.1016/0167-0115(86)90075-3 3763959

[B45] Kamal AM Salama OA El Saied KM Changes in amino acids profile and camel milk protein during the early lactation Int J Dairy Sci 2007 2 226 234

[B46] Mohamed BE Idam NZ Effect of camel milk on plasma lipid profile of hypercholesteremic rats OJVRTM 2011 15 314 317

[B47] Alhomida AS Junaid MA A-Jafari AA Total, free, short-chain and long-chain acyl carnitine levels in Arabian camel’s milk (Camelus dromedarius) J Ocul Pharmacol Ther 1997 13 381 387 10.1089/jop.1997.13.381 9261773

[B48] Karanth J Jeevaratnam K Effect of dietary lipid, carnitine and exercise on lipid profile in rat blood, liver and muscle Indian J Exp Biol 2009 47 748 753 19957888

[B49] Ziamajidi N Khaghani S Hassanzadeh G Vardasbi S Ahmadian S Nowrouzi A Ghaffari SM Abdirad A Amelioration by chicory seed extract of diabetes- and oleic acid-induced non-alcoholic fatty liver disease (NAFLD)/non-alcoholic steatohepatitis (NASH) via modulation of PPARα and SREBP-1 Food Chem Toxicol 2013 58 198 209 23603006 10.1016/j.fct.2013.04.018

[B50] Argo CK Northup PG Al-Osaimi AM Caldwell SH Systematic review of risk factors for fibrosis progression in non-alcoholic steatohepatitis J Hepatol 2009 51 371 379 10.1016/j.jhep.2009.03.019 19501928

[B51] Barbagallo M Effects of vitamin E and glutathione on glucose metabolism: Role of magnesium Hypertension 1999 34 1002 1006 10.1161/01.HYP.34.4.1002 10523398

[B52] Salami M Moosavi-Movahedi AA Ehsani MR Yousefi R Haertlé T Jean-Marc Chobert JM Razavi SH Henrich R Balalaie S Ebadi SA Pourtakdoost S Niasari-Naslaji A Improvement of the antimicrobial and antioxidant activities of camel and bovine whey proteins by limited proteolysis J Agric Food Chem 2010 58 6 3297 3302 10.1021/jf9033283 20175528

[B53] Yakaryilmaz F Guliter S Savas B Erdem O Ersoy R Erden E Akyol G Bozkaya H Ozenirler S Effects of vitamin E treatment on peroxisome proliferator-activated receptor alpha expression and insulin resistance in patients with non-alcoholic steatohepatitis: results of a pilot study Intern Med J 2007 37 229 235 10.1111/j.1445-5994.2006.01295.x 17388862

[B54] Al-Fartosi KG Khuon OS Al-Tae HI Protective role of camel's milk against paracetamol induced hepatotoxicity in male rats JPBMS 2011 2 1795 1799

[B55] Darwish HA Abd Raboh NR Mahdy A Camel's milk alleviates alcohol-induced liver injury in rats Food Chem Toxicol 2012 50 5 1377 83 10.1016/j.fct.2012.01.016 22281157

[B56] Wangoha J Faraha Z Puhana Z Iso-electric focusing of camel milk proteins Int Dairy J 1998 8 617 621 10.1016/S0958-6946(98)00092-2

[B57] Abd El-Mageed NM Hepatoprotective effect of feeding celery leaves mixed with chicory leaves and barley grains to hypercholesterolemic rats Pharmacogn Mag 2011 7 26 151 156 10.4103/0973-1296.80675 21716923 PMC3113355

[B58] Luo Q Cai Y Yan J Sun M Corke H Hypoglycemic and hypolipidemic effects and antioxidant activity of fruit extracts from Lycium barbarum Life Sci 2004 26 2 137 149 15519360 10.1016/j.lfs.2004.04.056

[B59] Baumrucker CR Errondu NE Insulin like growth factor (IGF) system in the bovine mammary gland and milk J Mammary Gland Biol Neoplasia 2000 5 53 55 10.1023/A:1009515232450 10791768

